# Clinical characteristics and vaccine effectiveness against SARS-CoV-2 Omicron subvariant BA.2 in the children

**DOI:** 10.1038/s41392-022-01023-w

**Published:** 2022-06-28

**Authors:** Xiaohe Li, Liwen Wu, Youzhi Qu, Mengli Cao, Jiaqi Feng, Hua Huang, Yi Liu, Hongzhou Lu, Quanying Liu, Yingxia Liu

**Affiliations:** 1 Shenzhen Key Laboratory of Pathogen and Immunity, National Clinical Research Center for Infectious Disease, State Key Discipline of Infectious Disease, Shenzhen Third People’s Hospital, Second Hospital Affiliated to Southern University of Science and Technology, No. 29, Bulan Road, Longgang District, Shenzhen, 518112 P. R. China; 2 School of Medicine, Southern University of Science and Technology, Shenzhen, 518055 P. R. China; 3 Shenzhen Key Laboratory of Smart Healthcare Engineering, Department of Biomedical Engineering, Southern University of Science and Technology, No. 1088, Xueyuan Rd., Xili, Nanshan District, Shenzhen, Guangdong 518055 P. R. China; 4 Department of Radiology, Shenzhen Third People’s Hospital, the second affiliated hospital of Southern University of Science and Technology, National Clinical Research Center for Infectious Diseases. No. 29, Bulan Road, Longgang District, Shenzhen, 518112 P. R. China

**Keywords:** Infectious diseases, Infection, Vaccines

**Dear Editor**,

The Omicron variant of severe acute respiratory syndrome coronavirus 2 (SARS-CoV-2) has rapidly replaced the Delta variant as a dominating SARS-CoV-2 variant because of natural selection, which favors the variant with higher infectivity and stronger vaccine breakthrough ability.^1^ Among three lineages BA.1 (B.1.1.529.1), BA.2 (B.1.1.529.2), and BA.3 (B.1.1.529.3) of Omicron, BA.2 is rising rapidly worldwide since January 2022 and become a new dominating “variant of concern” in China.^2^ With the current surge in Omicron in China, both the absolute number of children hospitalized with COVID-19 and the percentage of total COVID-19 hospitalizations have been boosting recently. Understanding the clinical characteristics of SARS-CoV-2 Omicron subvariant BA.2 in children and the protective effect of vaccines is of great help for epidemic prevention strategies. However, so far no sufficient experimental data have been reported about infectivity, disease severity, vaccine breakthrough capability, and antibody resistance from pediatric BA.2.

In this report, we included a total of 514 pediatric patients aged ≤14 years infected with SARS-CoV-2 admitted to Shenzhen Third People’s Hospital from January 11, 2020 to March 23, 2022, which account for 14% of all 3645 patients. There were 465 pediatric cases infected with Omicron variant BA.2, accounting for 18.4% (465/2534) of total BA.2 patients during the same period. The proportion of pediatrics infected with Omicron strain was significantly higher than that with Wild-type (4.8%, 41/853) or Delta variant (3.1%, 8/258) (Fig. 1a). Why is the incidence of Omicron variant BA.2 significantly higher in children than in non-Omicron patients? It has been reported that the transmission power of the Omicron variant is 2.8 times that of the Delta variant.^1^ Our result is in line with this view, indicating that 84.1% (391/465) of Omicron-infected children had multiple morbidity in family members, which is higher than the non-Omicron strains (75.6% for Wild-type and 50% for Delta strains). Our study also confirmed that the median incubation period of BA.2-infected children is only 1 day, which is shorter than non-Omicron infected children (Fig. 1b and Supplementary Table 1). The familial clustering may be associated with the greater infectivity of the Omicron BA.2 variant. In addition, there is a significant difference in the distribution of disease severity between the children in the Omicron BA.2 group (95.3% of mild disease) and the non-Omicron group (51.0% of mild disease) (*p* < 0.001). Children in the Omicron BA.2 group have a higher rate of fever (58.9% vs 34.7%, *p* = 0.001) and sore throat (30.0% vs 7.9%, *p* = 0.004) compared with children in the non-Omicron group, suggesting that children infected with Omicron BA.2 are more likely to have symptoms of fever and sore throat but less likely to develop pneumonia, which is consistent with previous reports.^3^

**Fig. 1:**
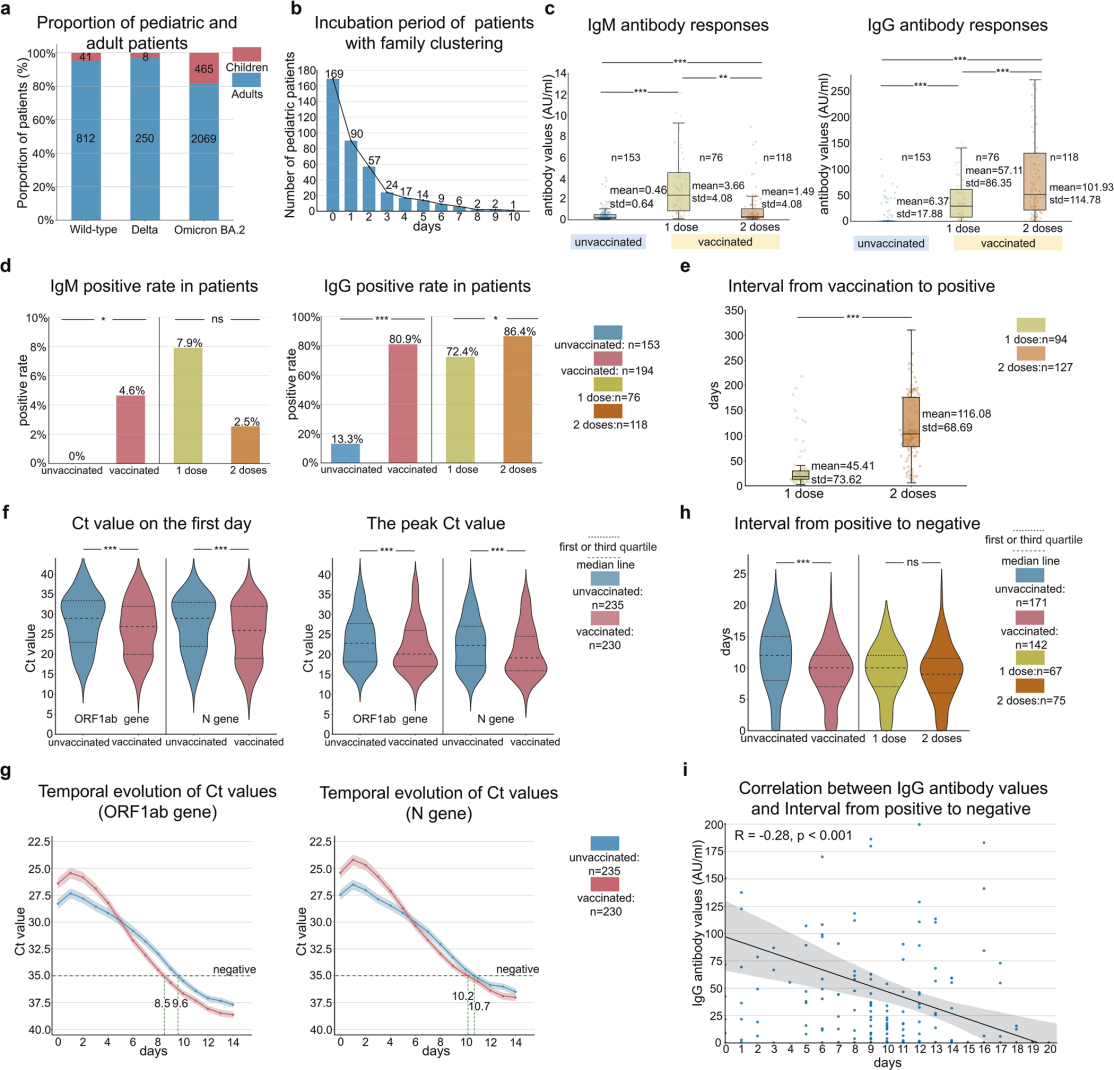
Characteristics and vaccine effectiveness against SARS-CoV-2 Omicron subvariant BA.2 in the children. **a** Proportion of pediatric and adult patients for Wild-type, Delta, and Omicron BA.2 strains. The red color represents pediatric patients, the blue color represents adult patients and the numbers in the bar represent the total number of patients in each category. **b** Incubation period of 391 pediatric patients with family clustering in Omicron BA.2 group. The incubation period is the time interval between the date of symptom onset in children and the date their family members first tested positive. **c** Antibody responses in the pediatric patients infected with Omicron BA.2. The left panel of **c** represents IgM antibody values (AU/ml) in vaccinated and unvaccinated patients. The right panel of **c** represents IgG antibody values (AU/ml) in vaccinated and unvaccinated patients. **p < 0.01, ***p < 0.001, Student’s *t*-test. **d** IgM and IgG positive rates in vaccinated and unvaccinated patients. Patients are IgM or IgG positive if the IgM or IgG antibody value is greater than 10 AU/ml. *p < 0.05, ***p < 0.001, ns indicates no significance, Student’s *t*-test. **e** The time interval from the last dose of vaccination to positive in vaccinated patients with 1 dose and 2 doses. ***p < 0.001, Student’s *t*-test. **f** Ct value (ORF1ab and N genes) in patients. The left panel of **f** represents Ct value (ORF1ab and N genes) on the first day in vaccinated and unvaccinated patients. The right panel of **f** represents the peak Ct value (ORF1ab and N genes) in vaccinated and unvaccinated patients. ***p < 0.001, Student’s *t*-test. **h** The time interval from the positive to negative in vaccinated and unvaccinated patients. ***p < 0.001, ns indicates no significance, Student’s *t*-test. **g** The temporal evolution of Ct value (ORF1ab and N genes) of vaccinated and unvaccinated groups. Patients in the unvaccinated and vaccinated groups are selected who turned negative (Ct values of ORF1ab gene and N gene are both greater than 35) and had no subsequent positive return. **i** Correlation between IgG antibody values and the time for nucleic acid negative conversion (Pearson correlation coefficient, R = −0.28, *p* < 0.001)

To investigate the protective effect of vaccines on Omicron BA.2-infected children, we compared the clinical characteristics of SARS-CoV-2 Omicron subvariant BA.2 between the vaccinated and unvaccinated individuals (Supplementary Table 2). Of 465 cases infected with Omicron BA.2, 230 (49.5%) were vaccinated against COVID-19, of which 96 (41.7%) received 1 dose and 134 (58.3%) received more than 2 doses. Most of these children, 88.7% (204/230) were vaccinated with inactivated vaccines. All the vaccinee were over 3 years old, and 80.7% (186/230) were over 6 years old. Compared with unvaccinated cases, the vaccinee had lower rates of fever (45.7% vs 71.9%, *p* < 0.001), shorter duration of fever (1.75 vs 2.53 days, *p* < 0.001) and higher rates of sore throat (35.2% vs 21.7%, *p* = 0.002). Overall, the vaccinee had fewer systemic symptoms such as persistent high fever, but was more likely to develop upper respiratory tract symptoms.

We next compared the differences in IgM and IgG antibody values and positive rates of the vaccinated cases between 1-dose and 2-dose vaccination (Fig. 1c, d). The overall positive rate in vaccinated group was higher than in unvaccinated group (4.6% vs 0%, *p* < 0.05). Although the IgM value of the 1-dose vaccinee (3.66 AU/ml) was higher than that of the 2-dose vaccinee (1.49 AU/ml), there was no significant difference in positive rate between the two groups (7.9% vs 2.5%, *p* > 0.05). More significantly, the IgG value and the positive rate of the 2-dose vaccinee (101.93 AU/ml, 86.4%) were higher than the 1-dose vaccinee (57.11 AU/ml, 72.3%), and both were higher than those of the unvaccinated group (6.37 AU/ml, 13.3%). Notably, we also found that the interval between the last dose of vaccine and the first nucleic acid positivity was much longer in the 2-dose vaccinee than in the 1-dose vaccinee (82 vs 12 days, *p* < 0.001) (Fig. 1e), which may account for the higher IgM value in the 1-dose vaccinee. And these results indicate that the 2-dose vaccination can stimulate the immune system to produce higher levels of and longer-lasting IgG antibodies.

Meanwhile, we compared the effects of vaccine on the change of nucleic acid with the course of disease. In all children with BA.2, the cycle threshold (Ct) values for SARS-CoV-2 open reading frame 1ab (OFR1ab) and nucleocapsid protein (N) at the first positivity and the peak of nucleic acid of nasopharyngeal swab in the vaccinated group were lower than those in the unvaccinated group (Fig. 1f). We analyzed the time of nucleic acid of nasopharyngeal swab negative conversion in 313 patients with ORF1ab and N genes Ct values over 35 for two consecutive days (twice with an interval at least 24 h) and no subsequent positive conversion. Compared with unvaccinated children, the Ct values (ORF1ab and N genes) increased faster in vaccinated children (for ORF1ab, slope of Ct values = 0.60 vs 0.52, *p* < 0.001; for N, slope of Ct values = 0.57 vs 0.47, *p* < 0.001), resulting in the time of nucleic acid negative conversion significantly shorter (9.3 vs 11.2 days, *p* < 0.001) (Fig. 1g, h).

To further explore the differences in the severity and course of the disease between 2-dose and 1-dose vaccination, an antibody test on 195 COVID-19 vaccinatee showed 157 children with positive antibodies and the rest 38 children without antibodies. The further analysis demonstrated significant differences in the fever rate (42.0% vs 60.5%, *p* = 0.040) and the time required for viral nucleic acid to turn negative (8.7 vs 10.8 days, *p* = 0.034) between the ones with/without antibodies (Supplementary Table 3). Correlation analysis of IgG antibody values and the time for nucleic acid negative conversion by Pearson correlation coefficient showed a significantly negative correlation (correlation coefficient R = −0.28, *p* < 0.001) existed between them (Fig. 1i). It shows that the higher the IgG antibody value at admission, the shorter the time required for nucleic acid negative conversion, which can benefit from COVID-19 vaccination.

In summary, our data indicate that children are susceptible to Omicron subvariant BA.2 infection, but present less severe symptoms. More importantly, our data suggest that regular inactivated COVID-19 vaccines can have a certain protective effect on the severity of clinical manifestations and the rapid clearance of the virus after Omicron BA.2 infection by stimulating antibody production. But the protective effect of antibodies produced by the vaccine declined over time. Our previous study showed that regular and booster vaccination with inactivated vaccines enhance the neutralizing activity against Omicron variant both in the breakthrough infections and vaccine.^4^ Therefore, booster vaccination of children may be an effective protection measure in the future.^5^

## Supplementary information

Supplementary Materials for Clinical characteristics and vaccine effectiveness against SARS-CoV-2 Omicron subvariant BA.2 in the children

Ethical Review

## Data Availability

The data that support the findings of this study are available from the corresponding authors upon reasonable request.
