# Supplementary material for: Clinical characteristics and vaccine effectiveness against SARS-CoV-2 Omicron subvariant BA.2 in the children
Source: Signal Transduct Target Ther. 2022 Jun 28;7:203. doi: 10.1038/s41392-022-01023-w (PMC9240082; doi:10.1038/s41392-022-01023-w)
Supplement: Supplementary file 1 — Supplementary Materials for Clinical characteristics and vaccine effectiveness against SARS-CoV-2 Omicron subvariant BA.2 in the children [file 41392_2022_1023_MOESM1_ESM.docx]

Supplementary Materials for
**Clinical characteristics and vaccine effectiveness against SARS-CoV-2 Omicron subvariant BA.2 in the children**

Xiaohe Li,^1,§^ Liwen Wu,^1,2,§^ Youzhi Qu,^3,§^ Mengli Cao,^1^ Jiaqi Feng,^1^ Hua Huang,^4^ Yi Liu,^1,2^ Hongzhou Lu,^1,*^ Quanying Liu,^3,*^ and Yingxia Liu,^1,*^

^1^Shenzhen Key Laboratory of Pathogen and Immunity, National Clinical Research Center for Infectious Disease, State Key Discipline of Infectious Disease, Shenzhen Third

People’s Hospital, Second Hospital Affiliated to Southern University of Science and Technology, No. 29, Bulan Road, Longgang District, Shenzhen 518112, P. R. China

^2^School of Medicine, Southern University of Science and Technology, Shenzhen 518055,P. R. China

^3^Shenzhen Key Laboratory of Smart Healthcare Engineering, Department of Biomedical Engineering, Southern University of Science and Technology, No. 1088, Xueyuan Rd., Xili, Nanshan District, Shenzhen, Guangdong, 518055, P. R. China

^4^Department of Radiology, The Third People's Hospital of Shenzhen, the second affiliated hospital of Southern University of Science and Technology, National Clinical Research Center for Infectious Diseases. No. 29, Bulan Road, Longgang District, Shenzhen 518112, P. R. China

^§^These authors contributed equally

*Correspondence: [yingxialiu@hotmail.com](mailto:yingxialiu@hotmail.com) (Y.L.); liuqy@sustech.edu.cn (Q.L.); luhongzhou@szsy.sustech.edu.cn (H.L.)

**This PDF file includes:**

Materials and Methods

Tables S1-S3

**Materials and Methods**

**Data collection**

Clinical information included demographic data, epidemiologic history, pre-existing diseases, symptoms, and laboratory examination results during hospitalization were obtained by reviewing their recorders at different times from the hospital’s computerized medical record system.

**CT analysis**

Several non-contrast thin-section chest CT scans were performed for each patient. All thin-section CT images were analyzed by a experienced radiologist.

**Disease severity classification of children COVID-19 patients**

The severity of COVID-19 was graded according to the China National Health Commission Guidelines for Diagnosis and Treatment of SARS-CoV-2 infection. Laboratory confirmed patients with fever, respiratory manifestations without radiological findings indicative of pneumonia were considered as the mild cases. Laboratory confirmed patients with fever, respiratory manifestations and radiological findings indicative of pneumonia were considered as the moderate cases. Laboratory confirmed patients with any of the following conditions were considered to have severe COVID-19: 1) high fever last more than 3days, 2) respiratory distress with the exclusion of cry and fever (age specific reference ranges: < 2 months: respiration rate (RR) ≥ 60/min; 2~12 months: RR ≥ 50/min; > 5 years: RR ≥ 30/min), 3) resting oxygen saturation ≤ 93%, 4) assisted respiration, cyanosis or intermittent apnea, 5) somnolence, convulsions, 6) refusing to eat or difficult to feed, symptoms of  dehydration.

**Data analysis**

Continuous variables are expressed as median and interquartile range (IQR). Categorical variables are described as number (percentage). Univariate comparisons of continuous variables were performed with an independent Student t-test for normally distributed data; otherwise, with Mann-Whitney U test. Categorical variables were compared using χ2 test or Fisher’s exact tests. Multivariate comparisons of continuous variables were performed with a mean vector test using hotelling T-Square test statistic for normally distributed data. A *p*-value<0.05 is considered statistically significant. The Pearson correlation analysis was applied to quantify the linear correlation between two different parameters such as the IgG antibody values and the time for nucleic acid negative conversion. Pearson coefficients range from +1 to -1, with +1 representing a positive correlation, -1 representing a negative correlation, and 0 representing no relationship. A *p*-value<0.05 is considered statistically significant.

To investigate the effect of vaccine on Ct values, we compared the temporal evolution of Ct values in the unvaccinated and vaccinated groups. First, we selected the patients who turned negative (both N gene and O gene >35) and had no subsequent positive return. Then, we performed linear interpolation based on the Ct values recorded on different days to obtain the temporal evolution of Ct values from the first day to the last test. We calculated the mean and standard deviation of Ct values day by day in the unvaccinated group and the vaccinated group, respectively, and plotted the time course of Ct values. Linear regression analysis was applied to calculate the slope of the Ct value, and to compare the speed of negative conversion between the vaccinated and unvaccinated groups.

**Supplementary Table 1. Epidemiological and clinical features of children hospitalized with SARS-CoV-2 infections.**

| **Characteristics** | **SARS-CoV-2 cases in children** | | |
| --- | --- | --- | --- |
|  | **Non-Omicron**  **(N = 49)** | **Omicron BA.2**  **(N = 465)** | ***P value*** |
| **Median age (range)** | 7 (0.25, 14) | 6 (0.08, 14) | 0.646 |
| **Age subgroups** |  |  | 0.491 |
| 0~3 years | 11/49 (22.4%) | 93/465 (20.0%) |  |
| 3~6 years | 8/49 (16.3%) | 111/465 (23.9%) |  |
| 6~14 years | 30/49 (61.2%) | 261/465 (56.1%) |  |
| **Male** | 22/49 (44.9%) | 264/465 (56.8%) | 0.111 |
| **Median BMI (IQR)** | 16.76 (14.90, 18.17) | 16.67 (14.88, 19.40) | 0.716 |
| **Familial clustering** | 35/49 (71.4%) | 391/465 (84.1%) | 0.025 |
| Median incubation period (range) | 2 (0, 10) | 1 (1,10) | <0.001 |
| **Presenting symptoms** |  |  |  |
| Fever | 17/49 (34.7%) | 274/465 (58.9%) | 0.001 |
| Duration, days (mean, range) | 2.25 (1, 10) | 2.25 (1, 16) | 0.445 |
| Body temperature (peak), degrees Celsius (mean, range) | 38.5 (37.3, 39.7) | 39.00 (37.0, 42.0) | 0.056 |
| Cough | 17/49 (34.7%) | 171/465 (36.8%) | 0.774 |
| Expectoration | 8/49 (16.3%) | 87/465 (18.7%) | 0.683 |
| Fatigue**^+^** | 1/38 (2.6%) | 45/373 (12.1%) | 0.103 |
| New loss of taste or smell**^+^** | 0/38 (0%) | 12/373 (3.2%) | 0.613 |
| Nasal congestion | 5/49 (10.2%) | 58/465 (12.5%) | 0.645 |
| Runny nose | 7/49 (14.3%) | 84/465 (18.1%) | 0.510 |
| Sore throat**^+^** | 3/38 (7.9%) | 112/373 (30.0%) | 0.004 |
| Myalgia**^+^** | 1/38 (2.6%) | 10/373 (2.7%) | 1.000 |
| Diarrhea | 3/49 (6.1%) | 25/467 (5.4%) | 0.741 |
| Headache**^+^** | 2/38 (5.3%) | 18/373 (4.8%) | 0.706 |
| Chill | 1/49 (2.0%) | 11/465 (2.4%) | 1.000 |
| Nausea or vomiting | 1/49 (2.0%) | 33/465 (7.1%) | 0.235 |
| Dizziness**^+^** | 0/38 (0%) | 6/373 (1.6%) | 1.000 |
| Chest pain**^+^** | 1/38 (2.6%) | 3/373 (0.8%) | 0.323 |
| **Symptoms counts** |  |  |  |
| patients with symptoms | 27/49 (55.1%) | 380/465 (81.7%) | <0.001 |
| 1 | 6/49 (12.2%) | 106/465 (22.8%) | 0.089 |
| 2 | 11/49 (22.4%) | 112/465 (24.1%) | 0.798 |
| ＞2 | 10/49 (20.4%) | 162/465 (34.8%) | 0.042 |
| **Disease severity** |  |  |  |
| Severe | 0/49 (0%) | 0/465 (0%) |  |
| Moderate | 24/49 (49.0%) | 22/465 (4.7%) | <0.001 |
| Mild | 25/49 (51.0%) | 443/465 (95.3%) | <0.001 |
| **Laboratory results** |  |  |  |
| WBC (× 10^9^/L) | 6.84 (4.79, 9.56) | 5.33 (4.26, 6.97) | 0.001 |
| LYM (× 10^9^/L) | 2.68 (2.05, 4.29) | 1.74 (1.08, 2.58) | ＜0.001 |
| NEU (× 10^9^/L) | 2.70 (1.60, 4.48) | 2.74 (1.80, 2.58) | 0.911 |
| Leukopenia | 14/42 (33.3%) | 64/154 (41.6%) | 0.334 |
| Lymphopenia | 1/42 (2.4%) | 51/154 (33.1%) | ＜0.001 |
| PLT (× 10^9^/L) | 261 (233.5, 320) | 236 (198, 288) | 0.003 |
| HB (g/L) | 131 (124, 140) | 131 (124, 137) | 0.906 |
| AST (U/L) | 36 (23.5, 46.25) | 36.05 (28.65, 44.25) | 0.354 |
| ALT (U/L) | 16 (12, 26.85) | 15 (12, 19) | 0.124 |
| CK (U/L) | 87 (67, 112.5) | 91 (70, 126) | 0.346 |
| CK-MB (ng/ml) | 0.83 (0.36, 2.44) | 0.38 (0.22, 0.74) | <0.001 |
| LDH (U/L) | 253 (200, 381) | 223.5 (194.75, 256) | 0.002 |
| ALB (g/L) | 45.6 (42.85, 47.45) | 45.25 (43.4, 47.1) | 0.858 |
| IL-6 (pg/ml) | 2.07 (1.49, 4.68) | 7.4 (2.71, 13.8) | ＜0.001 |
| CRP (mg/L) | 1.28 (0.37, 4.77) | 1.46 (0.40, 4.91) | 0.502 |
| PCT (ng/mL) | 0.06 (0.03, 0.15) | 0.08 (0.04, 0.16) | 0.334 |
| ESR(mm/h) | 10 (5, 15) | 6 (5, 9) | 0.003 |
| TroponinⅠ( μg/L ) | 0.0119 (0.0059, 0.0119) | 0.0119 (0.0119, 0.0119) | ＜0.001 |
| Bun (mmol/L) | 4.29 (3.41, 4.87) | 4.05 (3.52, 4.77) | 0.369 |
| Cr（μmol/L） | 32.8 (26.4, 41.8) | 42.1 (34.3, 50.1) | ＜0.001 |
| CD4 cells (count/μL) | 854 (709.75, 1577.5) | 659 (525, 1074) | 0.003 |
| CD8 cells (count/μL) | 767 (545, 1058.25) | 526 (367.5, 855) | 0.004 |
| **Image Features of CT scan** |  |  |  |
| Abnormal | 24/49 (49.0%) | 22/465 (4.7%) | <0.001 |
| Peripheral lung distribution | 21/24 (87.5%) | 20/22 (90.9%) | 1.000 |
| Unilateral involvement | 13/24 (54.2%) | 21/22 (95.5%) | 0.001 |
| Bilateral involvement | 11/24 (45.8%) | 1/22 (4.5%) | 0.001 |
| Rounded morphology | 13/24 (54.2%) | 5/22 (22.7%) | 0.029 |
| Patchy | 11/24 (40.9%) | 17/22 (77.3%) | 0.029 |
| Ground-glass opacities | 9/24 (37.5%) | 16/22 (72.7%) | 0.017 |
| Consolidation | 13/24 (54.2%) | 2/22 (9.1%) | 0.001 |
| Ground-glass opacities and consolidation | 5/24 (20.8%) | 4/22 (18.2%) | 1.000 |
| Interlobular septal thickening | 0/24 (0%) | 4/22 (18.2%) | 0.045 |

Data are presented as n/N (percentage %) or median (inter-quartile range [IQR]), unless otherwise indicated. N is the total number of patients with available data.p values were calculated by Mann-Whitney *U* test, *χ2* test or Fisher’s exact test, as appropriate.**^+^** Subjects were children over 3 years old. Leukopenia (× 10^9^/L): 2 months-2 years: <5, >2 years: <4; Lymphopenia (× 10^9^/L): 2-11 months: <4.0, 1-11 years: <1.5, 12+ years: <1

WBC: white blood cell; LYM: lymphocyte; NEU: neutrophil; PLT: platelet; HB: hemoglobin; AST: aspartate aminotransferase; ALT: alanine aminotransferase; CK: creatine kinase; CK-MB: CreatineKinase-MB; LDH: lactate dehydrogenase; ALB: albumin; IL-6: interleukin-6; CRP: C-reactive protein; PCT: procalcitonin; ESR: erythrocyte sedimentation rate; Bun: blood urea nitrogen; Cr: creatinine.

**Supplementary Table 2. Characteristics of children with Omicron BA.2 infections.**

| **Characteristics** | **Children with Omicron BA.2** | | **P value** |
| --- | --- | --- | --- |
|  | **Vaccinated**  **(N =230, 49.5%)** | **Unvaccinated**  **(N = 235, 50.5%)** |  |
| **Median age (range)** | 9 (3, 14) | 3 (0.08, 13) | <0.001 |
| **Age subgroups** |  |  | <0.001 |
| 0~3 years | 0/230 (0%) | 93/235 (39.6%) | <0.001 |
| 3~6 years | 44/230 (19.1%) | 67/235 (28.5%) | 0.013 |
| 6~14 years | 186/230 (80.9%) | 75/235 (31.9%) | <0.001 |
| **Median BMI (IQR)** | 17.28 (15.1, 19.95) | 16.2 (14.81, 18.67) | 0.052 |
| **Presenting symptoms** |  |  |  |
| Fever | 105/230 (45.7%) | 169/235 (71.9%) | <0.001 |
| Duration, days (mean, range) | 1.75 (1, 5) | 2.53 (1, 16) | <0.001 |
| Body temperature (peak), degrees Celsius (mean, range) | 38.8 (37.0, 42.0) | 39.1 (37.4, 40.8) | 0.002 |
| Cough | 89/230 (38.7%) | 82/235 (34.9%) | 0.395 |
| Expectoration | 46/230 (20.0%) | 41/235 (17.4%) | 0.480 |
| Fatigue**^+^** | 29/230 (12.6%) | 16/143 (11.2%) | 0.682 |
| New loss of taste or smell**^+^** | 7/230 (3.0%) | 5/143 (3.5%) | 0.773 |
| Nasal congestion | 32/230 (13.9%) | 26/235 (11.1%) | 0.353 |
| Runny nose | 45/230 (19.6%) | 39/235 (16.6%) | 0.405 |
| Sore throat**^+^** | 81/230 (35.2%) | 31/143 (21.7%) | 0.006 |
| Myalgia**^+^** | 8/230 (3.5%) | 2/143 (1.4%) | 0.329 |
| Diarrhea | 8/230 (3.5%) | 17/235 (7.2%) | 0.073 |
| Headache**^+^** | 13/230 (5.7%) | 5/143 (3.5%) | 0.345 |
| Chill | 4/230 (1.7%) | 7/235 (3.0%) | 0.379 |
| Nausea or vomiting | 12/230 (5.2%) | 21/235 (8.9%) | 0.118 |
| Dizziness**^+^** | 3/230 (1.3%) | 3/143 (2.1%) | 0.679 |
| Chest pain**^+^** | 0/230 (0%) | 3/143 (2.1%) | 0.056 |
| **Disease severity** |  |  |  |
| Moderate | 11/230 (4.8%) | 11/235 (4.7%) | 0.959 |
| Mild | 219/230 (95.2%) | 224/235 (95.3%) | 0.959 |
| **Imaging features （CT scan）** |  |  |  |
| Abnormal | 11/230 (4.8%) | 11/235 (4.7%) | 0.959 |

Data are presented as n/N (percentage %) or median (inter-quartile range [IQR]), unless otherwise indicated. N is the total number of patients with available data.

p values were calculated by Mann-Whitney *U* test, *χ2* test or Fisher’s exact test, as appropriate.

**^+^** Subjects were children over 3 years old.

**Supplementary Table 3. Clinical features of vaccinated children with Omicron BA.2 infections.**

| **Characteristics** | **Vaccinated**  **Children with Omicron BA.2** | | **P value** |
| --- | --- | --- | --- |
|  | **IgG positive**  **(N =157/195, 80.5%)** | **IgG negative**  **(N = 38/195, 19.5%)** |  |
| **Presenting symptoms** |  |  |  |
| Fever | 66/157 (42.0%) | 23/38 (60.5%) | 0.040 |
| Duration, days (mean, range) | 1.67 (1, 5) | 2.05 (1, 5) | 0.065 |
| Body temperature (peak), degrees Celsius (mean, range) | 38.7 (37.4, 40.2) | 39.1 (37.3, 42.0) | 0.106 |
| Sore throat**^+^** | 53/157 (33.8%) | 20/38 (52.6%) | 0.031 |
| **nucleic acid negative conversion time, days (mean, range)** | 8.73 (0, 18) | 10.77 (0, 17) | 0.034 |
| **Vaccine doses** |  |  | 0.012 |
| 1 | 56/157(35.7%) | 22/38 (57.9%) |  |
| 2 | 101/157 (64.3%) | 16/38 (42.1%) |  |

Data are presented as n/N (percentage %) or mean (range). N is the total number of patients with available data.

p values were calculated by Mann-Whitney *U* test, *χ2* test or Fisher’s exact test, as appropriate.

**^+^** Subjects were children over 3 years old.
