# Supplementary material for: Clinical characteristics and vaccine effectiveness against SARS-CoV-2 Omicron subvariant BA.2 in the children
Source: Signal Transduct Target Ther. 2022 Jun 28;7:203. doi: 10.1038/s41392-022-01023-w (PMC9240082; doi:10.1038/s41392-022-01023-w)
Supplement: Supplementary file 2 — Ethical Review [file 41392_2022_1023_MOESM2_ESM.pdf]

# 深圳市第三人民医院科研项目伦理审查意见书

受理号：深圳三医伦审科研申报字[ 2020-（013）]号

<table>
<tr><td>项目名称</td><td colspan="3">新型冠状病毒感染肺炎临床诊疗标准研究</td></tr>
<tr><td>申请类型</td><td colspan="3">深圳市科创委</td></tr>
<tr><td>申请科室</td><td>感染科</td><td>申请人</td><td>刘映霞</td></tr>
<tr><td>审查方式</td><td>快速审查</td><td>审查时间</td><td>2020 年 2 月 3 日</td></tr>
<tr><td rowspan="2">审查意见</td><td colspan="3">1.根据世界卫生组织、国家药品监督管理总局、广东省药学会等发布的最新版的《中华人民共和国药品管理法》，《药品临床试验管理规范》，《生物医学研究审查伦理委员会操作指南》，《人体生物医学研究国际伦理指南》，《药物临床试验机构伦理委员会操作规程》， 《药物临床试验伦理审查工作指导原则》，《药物临床研究伦理审查管理规范》，《医疗器械注册管理办法》，《影像医疗器械临床试验实用手册》，《药物临床试验质量管理规范》，《医疗器械临床试验质量管理规范》，《涉及人的生物医学研究伦理审查办法》，《体外诊断试剂注册管理办法》，《药物临床试验广东共识》，《医疗器械监督管理条例》，《医疗器械临床试验规定》，《实验动物管理条例》（2017年修订版）、《关于善待实验动物指导性意见》、《实验动物 福利伦理审查指南》和《实验动物 动物实验通用要求》等规定和要求<br>经审查，综上评价，意见如下：<br>☑同意 ☐作必要的修正后同意 ☐作必要的修正后重审<br>☐不同意 ☐终止或暂停已经批准的临床试验<br>其他具体意见：<br>2.课题立项后一月内需到伦理委员会备案，递交相关材料，进行伦理评审后获得伦理批件，便于伦理委员会对该项目质量、进展进行监督管理。</td></tr>
<tr><td colspan="3">深圳市第三人民医院医学伦理委员会<br><br>主审委员（签名）：[signature]<br><br>主任/副主任委员（签名）：[signature]<br>[stamp: 深圳市第三人民医院 科研伦理委员会]<br><br>2020 年 2 月 4 日</td></tr>
</table>





扫描全能王 创建
